# Multi-institutional prospective observational study of radiotherapy for metastatic bone tumor

**DOI:** 10.1093/jrr/rrae060

**Published:** 2024-08-20

**Authors:** Hideyuki Harada, Naoto Shikama, Akifumi Notsu, Hiroki Shirato, Kazunari Yamada, Haruka Uezono, Yutaro Koide, Hikaru Kubota, Takuya Yamazaki, Kei Ito, Joichi Heianna, Yukinori Okada, Ayako Tonari, Norio Katoh, Hitoshi Wada, Yasuo Ejima, Kayo Yoshida, Takashi Kosugi, Shigeo Takahashi, Takafumi Komiyama, Nobue Uchida, Misako Miwa, Miho Watanabe, Hisayasu Nagakura, Hiroko Ikeda, Tetsuo Saito, Isao Asakawa, Takeo Takahashi, Naoyuki Shigematsu

**Affiliations:** Division of Radiation Therapy, Radiation and Proton Therapy Center, Shizuoka Cancer Center, 1007 Shimonagakubo, Nagaizumi-cho, Sunto-gun, Shizuoka, 411-8777, Japan; Department of Radiation Oncology, Juntendo University Graduate School of Medicine, 2-1-1 Hongo, Bunkyo-ku, Tokyo, 113-8421, Japan; Clinical Research Center, Shizuoka Cancer Center, 1007 Shimonagakubo, Nagaizumi-cho, Sunto-gun, Shizuoka, 411-8777, Japan; Global Center for Biomedical Science and Engineering, Faculty of Medicine, Hokkaido University, Kita 15, Nishi 7, Kita-ku, Sapporo-shi, Hokkaido, 060-8638, Japan; Department of Radiation Oncology, Seirei Mikatahara General Hospital, 3453 Mikatahara, Chuo-ku, Hamamatsu-shi, Shizuoka, 433-8558, Japan; Department of Radiation Oncology, Hyogo Cancer Center, 13-70, Kitaojicho Akashi-shi, Hyogo, 673-0021, Japan; Department of Radiation Oncology, Aichi Cancer Center Hospital, 1-1 Kanokoden, Chikusa-ku, Nagoya-shi, Aichi, 464-8681, Japan; Division of Radiation Oncology, Kobe University Hospital, 7-5-2 Kusunoki-cho, Chuo-ku, Kobe-shi, Hyogo, 650-0017, Japan; Department of Radiology, Nagasaki University Hospital, 1-7-1 Sakamoto, Nagasaki-shi, Nagasaki, 852-8501, Japan; Department of Radiation Oncology, Tokyo Metropolitan Cancer and Infectious Diseases Center, Komagome Hospital, 3-18-22 Honkomagome, Bunkyo-ku, Tokyo, 113-8677, Japan; Department of Radiation Oncology, Nanbu Tokushukai Hospital, 171-1 Hokama, Yaese-cho, Shimajiri-gun, Okinawa, 901-0493, Japan; Department of Radiation Oncology, St. Marianna University School of Medicine, 2-16-1 Sugao, Miyamae-ku, Kawasaki-shi, Kanagawa, 216-8511, Japan; Department of Radiation Oncology, Kyorin University Hospital, 6-20-2 Shinkawa, Mitaka-shi, Tokyo, 181-8611, Japan; Department of Radiation Oncology, Hokkaido University Faculty of Medicine, Kita 15, Nishi 7, Kita-ku, Sapporo-shi, Hokkaido, 060-8638, Japan; Department of Radiation Oncology, Southern TOHOKU Proton Therapy Center, 7-172 Yatsuyamada, Koriyama-shi, Fukushima, 963-8052, Japan; Department of Radiology, Dokkyo Medical University, 880 Kitakobayashi, Mibu-machi, Shimotsuga-gun, Tochigi, 321-0293, Japan; Department of Radiology, Keio University School of Medicine, 5 Shinanomachi, Shinjuku-ku, Tokyo, 160-8582, Japan; Department of Radiation Oncology, Fujieda Municipal General Hospital, 4-1-11 Surugadai, Fujieda-shi, Shizuoka, 426-8677, Japan; Department of Radiation Oncology, Kagawa University Hospital, 1750-1 Ikenobe, Miki, Kita-gun, Kagawa, 761-0793, Japan; Department of Radiology, University of Yamanashi, 1110 Shimokato, Chuo-shi, Yamanashi, 409-3898, Japan; Department of Radiation Oncology, Tokyo Saiseikai Central Hospital, 1-4-17 Mita, Minato-ku, Tokyo, 108-0073, Japan; Department of Radiology, Sendai Kousei Hospital, 1-20 sutsumidori, Amemiya, Aoba-ku, Sendai-shi, Miyagi, 981-0914, Japan; Diagnostic Radiology and Radiation Oncology, Graduate School of Medicine, Chiba University, 1-8-1, Inohana, Chuo-ku, Chiba-shi, Chiba, 260-8670, Japan; Department of Radiology, KKR Sapporo Medical Center, Hiragishi Ichijo 6-3-40, Toyohira-ku, Sapporo-shi, Hokkaido, 062-0931, Japan; Department of Radiation Oncology, Osaka City General Hospital, 2 Chome-13-22 Miyakojimahondori, Miyakojima-ku, Osaka-shi, Osaka, 534-0021, Japan; Department of Radiation Oncology, Ariake Medical Center, 2600, Arao, Arao-shi, Kumamoto, 864-0041, Japan; Department of Radiation Oncology, Nara Medical University, 840 Shijo-cho, Kashihara-shi, Nara, 634-8522, Japan; Department of Radiation Oncology, Saitama Medical Center, Saitama Medical University, 1981 Kamoda, Kawagoe-shi, Saitama, 350-8550, Japan; Department of Radiology, Keio University School of Medicine, 5 Shinanomachi, Shinjuku-ku, Tokyo, 160-8582, Japan

**Keywords:** bone metastasis, prospective study, radiotherapy, pain relief, SRE

## Abstract

Purpose of this study is to evaluate patient characteristics, treatments and outcomes in bone metastasis radiotherapy practice. Patients for whom radiotherapy for bone metastasis was planned at 26 institutions in Japan between December 2020 and March 2021 were consecutively registered in this prospective, observational study. Study measures included patient characteristics, pain relief, skeletal-related events (SREs), overall survival and incidence of radiation-related adverse events. Pain was evaluated using a numerical rating scale (NRS) from 0 to 10. Irradiated dose was analyzed by the biologically effective dose (BED) assuming α/β = 10. Overall, 232 patients were registered; 224 patients and 302 lesions were fully analyzed. Eastern Cooperative Oncology Group Performance Status was 0/1/2/3/4 in 23%/38%/22%/13%/4%; 59% of patients had spinal metastases and 84% had painful lesions (NRS ≥ 2). BED was <20 Gy (in 27%), 20–30 Gy (24%), 30–40 Gy (36%) and ≥ 40 Gy (13%); 9% of patients were treated by stereotactic body radiotherapy. Grade 3 adverse events occurred in 4% and no grade 4–5 toxicity was reported. Pain relief was achieved in 52% at 2 months. BED is not related to pain relief. The cumulative incidence of SREs was 6.5% (95% confidence interval (CI) 3.1–9.9) at 6 months; no factors were significantly associated with SREs. With spinal lesions, 18% of patients were not ambulatory at baseline and 50% of evaluable patients in this group could walk at 2 months. The 6-month overall survival rate was 70.2% (95% CI 64.2–76.9%). In conclusion, we report real-world details of radiotherapy in bone metastasis.

## INTRODUCTION

Radiotherapy plays an important role in the treatment of metastatic bone tumors. In addition to pain relief, radiotherapy is also used to improve symptoms in complicated cases such as patients with paraplegia due to spinal cord compression, to prevent bone events such as pathological fractures and paralysis, and to provide long-term local control in oligo-metastases. Numerous randomized controlled trials have demonstrated the efficacy of single irradiation for pain relief [1]. However, while systemic therapy significantly improves survival, the endpoints of these trials are often limited to pain relief at 1 to 3 months and complicated lesions are often excluded, creating a gap that physicians should comprehensively consider in practice. In fact, reports have described that radiotherapy is not an option for single irradiation in a variety of scenarios [2]. There is a paucity of data showing what kind of radiotherapy is given to what kind of subjects in actual clinical practice, and limited data have been reported on prospective studies of clinical outcomes. Therefore, we planned to conduct a multicenter prospective observational study mainly at Japanese Society for Radiation Oncology-accredited centers to clarify the actual conditions of radiotherapy for patients with bone metastases in Japan and the efficacy of the treatment; Clinical Trial Registration number: UMIN000042491.

## METHODS

Patients who underwent radiotherapy for bone metastases at 26 centers throughout Japan between December 2020 and March 2021 were included. Patients who met eligibility criteria were consecutively and prospectively enrolled until 10 cases were enrolled at each institution. Patients were followed for 6 months from the date of enrollment of the last case. The eligibility criteria were as follows: written consent to participate in the study had been obtained from the patient and radiation therapy was planned for metastatic bone tumor. Patients who were deemed by their physicians to be unsuitable for participation in the study were excluded.

Radiotherapy was administered at each facility as clinical practice, while the dose fractionation and method were left up to the treating physician. Adjuvant and supportive care were provided by the patient’s physician as needed, and there were no restrictions on post-treatment cancer therapy. We collected the following information at the end of irradiation: age, sex, and Eastern Cooperative Oncology Group Performance Status (ECOG-PS) as patient background at the time of enrollment and disease information, including the name of the primary disease, whether the primary tumor was under control, the site of the lesion other than that to be irradiated, the history of treatment for the primary disease, the Surgical Instability Neoplastic Score (SINS) [3] and Bilsky score [4] in the case of spinal metastases, and Mirels score [5]. We also collected information on radiotherapy, including irradiation method and dose, pre-treatment pain score (numerical value) as a symptom-related variable, elapsed time (numerical value) since the onset of pain due to radiotherapy lesions at the time of registration, and whether the patient had been reviewed by a cancer board specific for bone metastases. Follow-up data were collected on the following items: pain, presence or absence of bone events, ambulatory status and survival status at 2 and 6 months after the start of treatment. Skeletal-related events (SREs) were defined as pathological fracture, spinal cord paralysis, surgery on the bone and re-irradiation (if applicable, presence and date of each). Adverse events were evaluated using the Common Terminology Criteria for Adverse Events v5.0 (CTCAE v5.0). Quality of life (EQ-5D-5L, EORTC-PAL15 and BM22) and employment status were also investigated, but an analysis of employment status has already been reported elsewhere [6], and QOL is currently being analyzed and is not included in this report [7]. Numerical rating scale (NRS) score of pain at the region treated with radiation therapy anchored at 0 and 10 was recorded. Patients were asked to score the NRS to reflect the worst pain they had experienced within the last 3 days. Narcotic doses were converted to oral morphine doses. Patients who had experienced pain with an NRS of ≥2 at the start of treatment were evaluated for pain at 2 and 6 months after radiotherapy. Pain relief was defined as a decrease in NRS of ≥2 or a decrease in opioid use of ≥25% compared with that at baseline, and complete pain resolution was defined as NRS 0 without an increase in opioid use. An increase in NRS of ≥2 or an increase in opioid use of ≥25% was defined as worsening, and an absence of any of the above was defined as an unchanged status [8].

The study was performed in compliance with the tenets of the Declaration of Helsinki and in accordance with the explanation provided to the participants.

### Statistics

Categorical variables were summarized by count and percentage, and continuous variables by mean, median, standard deviation and range. Association between factors and pain relief was investigated by Fisher exact test. Cumulative probability of the bone-related events and their risk factors were investigated by the competing risk analysis [9]. Death was treated as a competing risk. Gray test was used to compare cumulative probabilities. Survival analysis with Kaplan–Meier method and Cox proportional hazard regression was conducted to investigate overall survival. Factors included in multivariate Cox regression analysis were chosen from known risk factors. Time to SREs and overall survival time were defined as the time from enrollment to occurrence of SREs and death, respectively. Two-sided *P*-values <0.05 were considered to be statistically significant. Analyses were performed with the use of the R statistical package, version 4.2.2 (R Core Team [2022], www.r-project.org).

## RESULTS

### Patient characteristics and treatment

Consort of this study was already reported [6]. Briefly, there were 333 patients with bone metastasis referred for radiation therapy in 26 centers during December 2020 to March 2021. Among them, 224 patients were analyzed in the study described in this report. The 2-month follow-up was conducted in 186 patients and the 6-month follow-up in 131 patients.

Patient characteristics, radiotherapy method and dose and cancer treatment after radiotherapy are summarized in Table 1. Overall, 60% of the patients were in good general condition with ECOG-PS 0–1, while 16% had a decrease in ECOG-PS to 3–4, which included patients with widely varying general conditions at the time of enrollment. In terms of primary disease, lung cancer and breast cancer accounted for 50% of the total. Regarding irradiation site, 52% of all cases were in the spine; painful bone metastases with NRS ≥2 accounted for 84% of all cases.

**Table 1:** Patient characteristics and treatment summary

Characteristics, *N* (%)		*N* = 224	
Age (year)		Radiation method	
Mean (SD)	68 (11)	Conventional	204 (91)
Median (Range)	70 (28–89)	SBRT/IMRT	20 (9)
Gender		Index pain	
Female	85 (38)	More than 0, N(%)	199(89)
Male	139 (62)	Pain score except for index pain	
ECOG-PS		More than 0, N(%)	98(44)
0	52 (23)	Cause of pain except for index pain	
1	86 (38)	Adverse event	2 (1)
2	50 (22)	Unknown	15 (7)
3	28 (13)	Tumor pain	69 (31)
4	8 (4)	Except for tumor pain	13 (6)
Primary site		SINS score	
Lung	80 (36)	Number of evaluated patients	132
Breast	33 (15)	Mean (SD)	8.8 (3.6)
Liver, bile duct, pancreas	20 (9)	Median (Range)	8 (1–18)
Kidney, Ureter	19 (8)	Bilsky grade	
Prostate	15 (7)	0 or 1	88 (39)
Colon	15 (7)	2	29 (13)
Unknown	8 (4)	3	13 (6)
Head and neck (except thyroid)	6 (3)	Not evaluated	94 (42)
Uterus	3 (1)	Mirels score	
Soft tissue or bone sarcoma	2 (1)	Number of evaluated patients	36
Thyroid	1 (0)	Mean (SD)	8.6 (1.7)
Others	22 (10)	Median (Range)	9 (3–12)
Controlled primary lesion		Walking status	
Yes	116 (52)	Possible	141 (63)
No	108 (48)	In room	57 (25)
Active metastasis except for irradiated lesion		Impossible	26 (12)
Regional lymph node	71 (32)	Opioid use	
Distant metastasis except bone	119 (53)	Yes	100 (45)
Bone metastasis	142 (63)	No	124 (55)
Oligometastasis		Oral morphine equivalent dose (mg/day)	
Yes	15 (7)	Number of evaluated patients	100
No	209 (93)	Mean (SD)	45.6 (62.2)
Previous cancer therapy		Median (Range)	30 (3–360)
Systemic therapy	154 (69)	BED10	
Surgery	106 (47)	Number of sites	302
Radiation site		<20 Gy	81 (27)
Pelvis (except for sacral spine)	65 (29)	20 Gy = <, <30 Gy	72 (24)
Lumbar spine	62 (28)	30 Gy = <, <40 Gy	108 (36)
Thoracic spine	58 (26)	40 Gy = <	41 (14)
Femoral bone	30 (13)	Radiation duration (days)	
Cervical spine	27 (12)	Median (Range)	7 (1–44)
Rib	19 (8)	Multidisciplinary cancer board discussion	
Scapula and clavicle	13 (6)	Yes	24 (11)
Sacral spine	8 (4)	No	200 (89)
Humerus	6 (3)	Post radiation cancer therapy (Start of treatment ~2 months)	
Skull	5 (2)	Systemic cancer treatment	135 (60)
Lower leg bone	4 (2)	BMA	95 (42)
Forearm bone	2 (1)	Post radiation cancer therapy (2~6 months)	
Others	3 (1)	Systemic cancer treatment	102 (46)
Previous local therapy		BMA	74 (33)
Radiotherapy	22 (10)		
Surgery	20 (9)		
Treatment setting			
Postoperative	14 (6)		
Preoperative	2 (1)		
Radiotherapy only	208 (93)		

SD = standard deviation, ECOG-PS = Eastern Cooperative Oncology Group Performance Status, SBRT = stereotactic body radiotherapy, IMRT = intensity-modulated radiation therapy, Index pain = pain from target lesions for radiotherapy, SINS = Surgical Instability Neoplastic Score, BED = biologically effective dose, BMA = bone-modifying agent

In terms of the irradiation method, 9% of the patients were treated with high-precision irradiation such as stereotactic body radiotherapy (SBRT) and intensity-modulated radiation therapy (IMRT), while the remaining 91% were treated with conventional irradiation. Overall, 10% of the patients were irradiated as re-irradiation. Of the 26 centers, 14 (54%) had at least one case treated with high-precision irradiation such as SBRT or IMRT and 13 (50%) had at least one re-irradiation case. Twenty-four (11%) of the cases were reviewed by a cancer board dedicated to bone metastases.

For observational studies, actual radiotherapy was performed in various dose fractions, and the dose was evaluated using the biologically effective dose (BED10) with α/β = 10 [10]. The doses of 8 Gy/1 dose, 4 Gy/5 doses and 3 Gy/10 doses frequently used for bone metastases were 14.4, 28 and 39 Gy, respectively. The median BED by site was 28 Gy, and 84% of all patients underwent treatment with BED of <40 Gy using the conventional method. Among patients with an ECOG-PS of 3–4 at enrollment, 61% received BED of <30 Gy with the conventional method, while among patients with an ECOG-PS of 0–1, 41% received BED of <30 Gy with the conventional method (*P* = 0.08, Supplemental Table 1). The results indicated that irradiation method and dose fractionation were tended to be selected according to PS at the time of enrollment.

### Pain relief

**Fig. 1:**
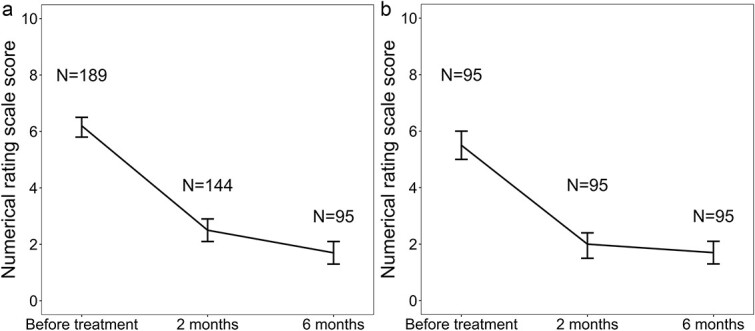
NRS score of pain at the region treated with radiation therapy. The bars represent mean and its 95% CI. (**a**) Pain scores in patients who were evaluable before treatment and at 2 and 6 months after initiation of the radiation therapy. (**b**) Pain score in patients who were evaluable 6 months after initiation of the radiation therapy. CI = confidence interval.

Pain was evaluated at 2 and 6 months after radiotherapy in 189 patients who had pain rated as NRS ≥2 at the start of treatment. Overall, 144 patients were evaluable at 2 months and 95 patients at 6 months. Of the evaluable patients, pain resolved or decreased in 75 (52%) at 2 months and in 55 (58%) at 6 months. Pain completely resolved in 32 patients (22%) after 2 months and 31 patients (33%) after 6 months. Pain was analyzed by dividing subjects according to baseline pain levels into mild, moderate and severe pain, which showed a pain-reducing effect regardless of the intensity of baseline pain (Table 2). The transition of pain score is shown in Fig. 1. Among 189 patients who had NRS ≥2 at the start of treatment, mean score (95% confidence interval;CI) was 6.2 (5.8–6.5), 2.5 (2.1–2.9) and 1.7 (1.3–2.1) at baseline at 2 and 6 months, respectively.Among 95 patients who were evaluable 6 months after initiation of the radiation therapy, mean score (95%CI) was 5.5 (5.0–6.0), 2.0 (1.5–2.4) and 1.7 (1.3–2.1) at baseline at 2 and 6 months, respectively. In patients with pain rated as NRS ≥2, systemic chemotherapy or hormone therapy was found to be associated with pain relief at 2 months, but no other factors showed any association (Table 3). Of the 17 patients who had pain of NRS ≥2 and were treated with re-irradiation, 10 (59%) had pain reduction or resolution after 2 months. Of the 127 patients who were not re-irradiated, pain had been reduced or eliminated in 65 (51%) after 2 months. There was no statistically significant difference between the two groups in this regard. Eighty-seven patients had severe pain with an NRS of 7 or greater at baseline. Of these, 63% were using opioid analgesics. At baseline, 100 of 224 patients (45%) were using opioids, but, at 2 and 6 months, 82 of 186 patients (44%) and 43 of 130 patients (33%), respectively, were using opioids. Of these 100 patients, 68 were evaluable for pain after 2 months. Of these, 26(38%) had a 25% or greater reduction in opioid use, and 25 had eliminated or reduced pain (Supplemental Table 2). There was no significant difference in pain relief (CR + PR) or CR (data not shown) between SBRT and conventional RT in this study.

**Table 2:** Pain relief for patients who experienced pain of NRS ≥2 at the start of treatment

	2 months					
NRS before treatment	N (%)	Complete pain resolution (CR)	Partial pain relief (PR)	Unchanged	Worsening	CR + PR (*P* = 0.689)
All (*N* = 189)	144	32 (22)	43 (30)	56 (39)	13 (9)	75 (52)
Mild (2 ~ 4, *N* = 51)	46	17 (37)	8 (17)	16 (35)	5 (11)	25 (54)
Moderate (5 ~ 6, N = 51)	39	8 (21)	10 (26)	16 (41)	5 (13)	18(47)
Severe (7 ~ 10, *N* = 87)	59	7 (12)	25 (42)	24 (41)	3 (5)	32(56)
	6 months					
NRS before treatment	N (%)	Complete pain resolution (CR)	Partial pain relief (PR)	Unchanged	Worsening	CR + PR (*P* = 0.111)
All (*N* = 189)	95	31 (33)	24 (25)	32 (34)	8 (8)	55(58)
Mild (2 ~ 4, *N* = 51)	40	13 (32)	5 (12)	15 (38)	7 (18)	18(44)
Moderate (5 ~ 6, N = 51)	24	8 (33)	8 (33)	8 (33)	0 (0)	16(66)
Severe (7 ~ 10, *N* = 87)	31	10 (32)	11 (35)	9 (29)	1 (3)	21(67)

NRS = numerical rating scale

Percentages were calculated using the number of evaluable cases at that time as the denominator.

**Table 3:** Factors associated with pain relief at 2 months after trial enrollment

Variable	N(%)	Pain relief (*N* = 75)	Unchanged or worsening (*N* = 78)	*P*-value
Primary site				0.394
Lung	57	25 (44)	32 (56)	
Breast	25	13 (52)	12 (48)	
Liver, bile duct, pancreas	12	6 (50)	6 (50)	
Kidney, urether	11	6 (55)	5 (45)	
Prostate	11	7 (64)	4 (36)	
Colon	9	7 (78)	2 (22)	
Unknown	6	4 (67)	2 (33)	
Head and neck (except thyroid)	5	1 (20)	4 (80)	
Uterus	2	0 (0)	2 (100)	
Soft tissue or bone sarcoma	2	0 (0)	2 (100)	
Thyroid	1	1 (100)	0 (0)	
Others	12	5 (42)	7 (58)	
Systemic chemotherapy or hormonal therapy				0.002
Yes	113	64 (57)	49 (43)	
No	40	11 (28)	29 (72)	
BMA				0.105
Yes	83	46 (55)	37 (45)	
No	70	29 (41)	41 (59)	
Radiation method				0.396
3DCRT	140	67 (48)	73 (52)	
SBRT/IMRT	13	8 (62)	5 (38)	
Previous Radiotherapy				0.447
Yes	17	10 (59)	7 (41)	
No	136	65 (48)	71 (52)	
Oligometastases				0.742
Yes	9	5 (56)	4 (44)	
No	144	70 (49)	74 (51)	
BED				0.439
Conventional, <20 Gy	37	19 (51)	18 (49)	
Conventional, > = 20 Gy, <30 Gy	36	13 (36)	23 (64)	
Conventional, > = 30 Gy, <40 Gy	54	30 (56)	24 (44)	
Conventional, > = 40 Gy	13	5 (38)	8 (62)	
SBRT/IMRT, <50 Gy	3	2 (67)	1 (33)	
SBRT/IMRT, > = 50 Gy	10	6 (60)	4 (40)	
Steroid use(baseline)				0.781
Yes	14	6 (43)	8 (57)	
No	139	69 (50)	70 (50)	

BMA = bone-modifying agent, BED = biologically effective dose, SBRT = stereotactic body radiotherapy, IMRT = intensity-modulated radiation therapy

### Skeletal-related events (SREs)

**Fig. 2:**
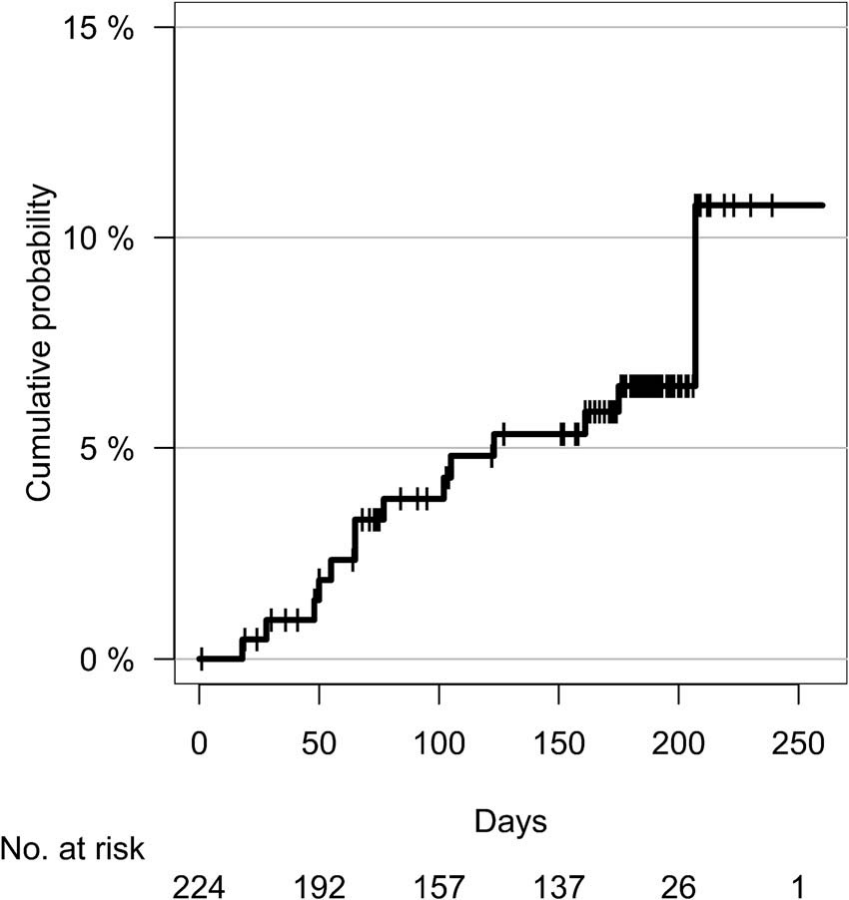
Cumulative probability of SREs. SREs = skeletal-related events (spinal cord paralysis, pathological fracture, surgery at the irradiated site or re-irradiation).

Of the 224 patients analyzed, SREs (spinal cord paralysis, pathological fracture, surgery at the irradiated site or re-irradiation) occurred in 14 patients (Supplemental Table 3), with 60- and 180-day cumulative probabilities of occurrence of 2.4% (95% confidence interval (CI) 0.3–4.4%) and 6.5% (95% CI 3.1–9.9%), respectively (Fig. 2). Re-irradiation was performed in eight patients (3.6%). Unfortunately, data have not been collected on which site SREs occurred when they occurred in patients who were irradiated in multiple sites. Therefore, if an SRE was reported during the follow-up of a patient who had been irradiated in the spine or extremities, the event was calculated as if it had occurred in each case.

Treatment for spinal metastases was performed in 132 cases. Of these, 42 patients (32%) had Bilsky grade 2–3 spinal cord compression of the tumor. Of the 25 patients who were ambulatory before radiotherapy, 19 were ambulatory 2 months later, 1 was not ambulatory, and 5 were not evaluable. Of the 17 patients who were not ambulatory before radiotherapy, 7 recovered their walking ability at 2 months, 4 did not and 6 could not be evaluated (Table 4).

**Table 4:** Change in ambulatory status for patients with spinal metastases

Baseline Bilsky score	Baseline ambulatory status	*N*	Ambulatory status at 2 months, *N* (%)			
			Ambulatory	In room	Not ambulatory	NE
All (*N* = 132)	All	132	78 (59)	20 (15)	8 (6)	26 (20)
	Ambulatory	79	62 (78)	8 (10)	0 (0)	9 (11)
	In room	29	12 (41)	9 (31)	1 (3)	7 (24)
	Not ambulatory	24	4 (17)	3 (12)	7 (29)	10 (42)
0 or 1 (*N* = 88)	All	88	56 (64)	14 (16)	3 (3)	15 (17)
	Ambulatory	61	48 (79)	7 (11)	0 (0)	6 (10)
	In room	20	8 (40)	7 (35)	0 (0)	5 (25)
	Not ambulatory	7	0 (0)	0 (0)	3 (43)	4 (57)
2 (*N* = 29)	All	29	14 (48)	5 (17)	3 (10)	7 (24)
	Ambulatory	11	8 (73)	1 (9)	0 (0)	2 (18)
	In room	5	2 (40)	2 (40)	0 (0)	1 (20)
	Not ambulatory	13	4 (31)	2 (15)	3 (23)	4 (31)
3 (*N* = 13)	All	13	6 (46)	1 (8)	2 (15)	4 (31)
	Ambulatory	5	4 (80)	0 (0)	0 (0)	1 (20)
	In room	4	2 (50)	0 (0)	1 (25)	1 (25)
	Not ambulatory	4	0 (0)	1 (25)	1 (25)	2 (50)

NE = not evaluable

The 60- and 180-day cumulative probabilities of bone-related events in all spinal metastases patients without paralysis or fracture at the start of radiotherapy were 2.1% (95% CI: 0.0–5.0%) and 3.3% (95% CI: 0.0–6.9%), respectively (Supplemental Fig. S1). Meanwhile, the incidences of SRE at 6 month by SINS of 0–6, 7–12 and 13–18 were 3.0% (95% CI: 0.0–9.0%), 3.1% (95% CI: 0.0–7.3%) and 4.5% (95% CI: 0.0–13.5%), respectively. Of the 32 limb cases, excluding those that were preoperatively or postoperatively irradiated or unable to walk at the time of enrollment, 4 had bone-related events. The 2- and 6-month cumulative SRE incidences were 3.1% and 12.8%, respectively (Supplemental Fig. S2).

### Survival period

**Fig. 3:**
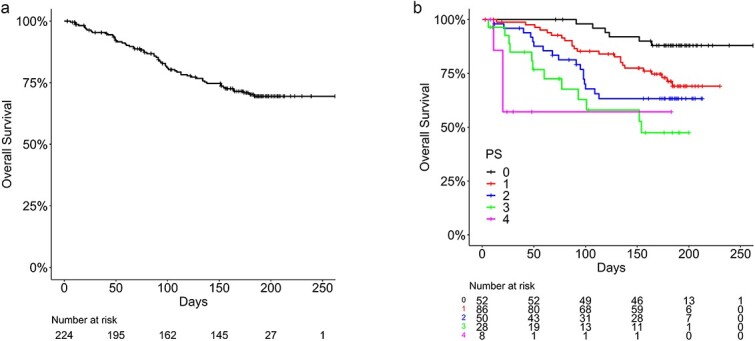
(**a**) Overall survival. (**b**) Overall survival by performance status. PS = Eastern Cooperative Oncology Group Performance Status.

Overall survival, assessed by the Kaplan–Meier method, did not reach the median, with 2- and 6-month survival rates of 90.2% (95% CI 86.3–94.3%) and 70.2% (95% CI 64.2–76.9%), respectively (Fig. 3a). Survival time differed according to baseline PS (*P* < 0.001) (Fig. 3b). Univariate and multivariate analyses of survival showed that the poor prognostic factors were poor PS, regional lymph node metastases, bone metastases except for irradiated bone and the primary tumor being hepatobiliary, pancreas or colorectal, while the good prognostic factor was the primary tumor being breast cancer (Table 5).

**Table 5:** Risk factors for overall survival

Variable	*N*	Univariate			Multivariate (*N* = 224)		
		HR	95%CI	*P*-value	HR	95%CI	*P*-value
Age							
<70 years	108	Reference			Reference		
> = 70 years	116	0.70	0.42–1.17	0.172	0.77	0.45–1.33	0.355
Sex							
Female	85	Reference			Reference		
Male	139	1.16	0.68–1.97	0.583	0.95	0.51–1.77	0.868
ECOG-PS							
0 or 1	138	Reference			Reference		
2	50	1.96	1.07–3.56	0.028	2.26	1.20–4.26	0.011
3 or 4	36	3.76	2.01–7.04	<0.001	3.61	1.82–7.19	<0.001
Primary site							
Lung	80	Reference			Reference		
Breast	33	0.24	0.06–1.05	0.057	0.21	0.05–1.00	0.050
Liver, bile duct, pancreas	20	2.78	1.28–6.02	0.010	3.63	1.55–8.53	0.003
Kideny, urether	19	1.36	0.50–3.66	0.545	1.56	0.56–4.37	0.394
Prostate	15	1.23	0.42–3.64	0.707	1.15	0.36–3.73	0.810
Colon	15	2.86	1.24–6.60	0.014	2.80	1.01–7.72	0.047
Others	42	1.71	0.85–3.43	0.134	1.72	0.79–3.74	0.174
Controlled primary lesion							
Yes	116	Reference			Reference		
No	108	1.07	0.65–1.77	0.795	0.93	0.47–1.84	0.834
Regional lymphnode metastass							
No	153	Reference			Reference		
Yes	71	1.61	0.97–2.69	0.067	1.88	1.04–3.38	0.036
Distant metastasis except for bone metastasis							
No	105	Reference			Reference		
Yes	119	1.57	0.93–2.65	0.091	1.06	0.56–2.01	0.852
Bone metastasis except for irradiated lesion							
No	82	Reference			Reference		
Yes	142	2.02	1.12–3.61	0.019	1.92	1.03–3.56	0.039
Systematic therapy before radiotherapy							
Yes	154	Reference			Reference		
No	70	0.97	0.56–1.69	0.924	0.93	0.49–1.76	0.819
Surgery before radiotherapy							
Yes	106	Reference			Reference		
No	118	1.03	0.62–1.70	0.918	0.85	0.41–1.77	0.664

HR = hazard ratio, CI = confidence interval, ECOG-PS = Eastern Cooperative Oncology Group Performance Status

### Adverse events

Adverse events were generally mild, with grade 3 lymphopenia occurring in five patients (2%), and fracture, ileus, pharyngeal mucositis and dysphagia in only one patient (0.4%) each.

## DISCUSSION

This study involved the prospective collection of data on radiotherapy for bone metastases, revealing the reality of treatment in the real world. Although the main role of radiotherapy for bone metastases is undoubtedly pain palliation, in some cases physicians administer radiotherapy with the intention of local control and reduction of bone-related events in addition to pain palliation. Overall, 84% of the patients in this study had painful bone metastases with NRS ≥2, indicating that the majority of patients were treated for pain relief.

The pain response rate for evaluable patients based on ICPRE is reported to be 60% [11], and this study also showed 52% pain relief at 2 months, with the pain-relieving effects replicating those previously reported [1, 11]. The present study was an observational study with wide-ranging eligibility criteria, and patients with various systemic conditions and complicated cases were also enrolled. Nevertheless, the pain-relieving effects reported in clinical trials were reproduced, indicating that the pain-relieving effects of radiotherapy are broadly applicable to patients with painful bone metastases. The pain-relieving effect after 2 months was better in patients who received post-irradiation systemic drug therapy (Table 3). To the best of our knowledge, there are no reports suggesting that systemic therapy after palliative radiotherapy may be effective in relieving pain from bone metastases. Although it is not intended to have a therapeutic effect on bone metastases, it may result in an enhanced antitumor effect on metastatic lesions and might provide pain relief by shrinking osteolytic lesions and promoting bone remodeling at the site of irradiation. This study showed that radiotherapy can be expected to provide pain relief even for bone metastases with high baseline pain (Table 2). Meanwhile, 44% of patients complained of pain from lesions that were not the radiotherapy target lesions, and more than half of these complaints involved tumor-related pain (Table 1). In these cases, radiotherapy alone is not sufficient to relieve pain, indicating that analgesics should be used in combination with radiotherapy. Although reports have been published stating that SBRT is more effective in relieving pain than conventional RT [12, 13], there was no significant difference in pain relief between SBRT and conventional RT in this study.

In this study, there were cases in which radiotherapy was applied to prevent the occurrence of symptoms and bone-related events. A recently reported randomized phase II trial of RT for asymptomatic bone metastases suggested that RT may be effective in preventing SRE [14]. The cumulative incidences of bone-related events in all patients were 2.4% at 2 months and 6.5% at 6 months, which is sufficiently low to be acceptable, although whether radiotherapy reduced the risk of bone-related events could not be evaluated because there was no group for comparison of the results. Meanwhile, only 17% of patients who were unable to walk due to spinal cord compression at the start of radiotherapy were able to walk 2 months after the start of irradiation. Unfortunately, data on the time between the onset of symptoms and the start of radiotherapy are not available, but they indicate the need to consider whether spinal surgery should be performed in each case. In this analysis, 76% of patients with Bilsky 2–3 compression who were able to undergo radiotherapy at the time they were able to walk were able to maintain ambulatory function 2 months later, suggesting that it is important to perform radiotherapy before symptoms appear in spinal metastases with spinal cord compression.

This study suggested associations of pretreatment ECOG-PS with BED and SBRT or IMRT use: more than half of PS4 patients with worsening PS chose a schedule with BED of <20 Gy. For PS3 patients, a short-course schedule of 8 Gy as a single fraction or 4 Gy administered 5 times was also selected by more than half of the patients. In contrast, >30% of the patients with ECOG-PS 0 received BED of 40 Gy or higher or SBRT, and this study also showed that survival varied depending on ECOG-PS, with a 6-month survival rate of >80% for PS0. This suggests that, in patients with good ECOG-PS, radiation oncologists choose dose fractionation with the expectation of a durable response, not just temporary pain relief. Indeed, while the 6-month survival rate exceeded 70%, re-irradiation was needed in 4% of the cases. This not only indicates that >70% of patients require a treatment effect that lasts longer than 6 months, not just a limited effect of 1–3 months, but also that the majority of patients did not need to be re-irradiated, suggesting that the dose selection was the result of a comprehensive judgment. The prognosis for colorectal cancer bone metastases was poorer than for lung cancer. The previous report analyzing prognostic factors also reported the median suvival time of 4.4 months for bone metastases from colorectal cancer, shorter than the 15.2 months for lung cancer patients who were indicated for treatment with molecularly targeted agents and the 4.8 months for other lung cancer [15]. Also, bone metastases except for irradiates lesions is a significant prognostic factor in this cohort. Although radiation therapy is usually administered to symptomatic sites, asymptomatic lesions are often present outside of the irradiated area. This result is considered consistent with the report that the existence of multiple metastases is reported as a significant prognostic factor in previous report [15].

In the cohort in this study, no SRE occurred in cases of oligo-metastases or in cases treated with SBRT or IMRT. Long-term follow-up data on spinal SBRT also indicate better local control with SBRT [16], and appropriate selection of cases is warranted. Some SBRT cases had BED of <50 Gy. The risk of recurrence may be a concern with a longer follow-up. It is hoped that the dose will be optimized in the future as guidelines for SBRT become more widely available.

To make appropriate clinical judgments, it is desirable for cases to be reviewed by a cancer board when the expected prognosis is long, when multidisciplinary treatment such as surgery and systemic therapy is necessary, and when high-precision treatment such as SBRT or IMRT is being considered. In the current study, only 11% of cases with bone metastases were actually reviewed by the cancer board, which should be improved in the future.

There are several limitations to this study. First, although data were collected from 26 facilities throughout Japan, all of them were board-certified facilities, which means that selection bias of the facilities could not be eliminated. In addition, the number of cases was limited because only 10 consecutive cases of radiotherapy for bone metastases were enrolled during the study period due to the burden on participating institutions. Another limitation is that, although follow-up data were obtained prospectively, follow-up data could not be obtained when the follow-up observation at the facility where the treatment was performed had been completed. In addition, although the 6-month survival rate was 70%, longer-term follow-up was not possible.

We believe that the findings of this study are valuable not only for pain relief, but also for prospective and comprehensive evaluation of radiotherapy for bone metastases. The effect of pain relief for bone metastases is well known and consisted with this study, but since it has been shown that SRE is less common in irradiated cases, it will be possible to explain less occurrence of SRE to patients based on this evidence. In conclusion, this work reveals that radiation oncologists are comprehensively evaluating patients with bone metastases and determining the actual radiotherapy regimen. The efficacy in relieving pain and preventing SRE shown in this prospective observational study was promising, indicating high efficacy and low adverse events associated with radiotherapy. These results may be useful for patients with bone metastases and physicians in making treatment choices in clinical practice. We believe that the prospective clinical data obtained here can be used as a benchmark for new intervention studies.

## Supplementary Material

Fig_S1_r3_rrae060

Fig_S2_r3_rrae060

supplemental_table_1_rrae060

Supplemental_Table2_rrae060

Supplemental_Table_3_rrae060

## PRESENTATION AT A CONFERENCE

Part of this study were presented at the 64th American Society for Radiation Oncology (ASTRO) annual meeting and the 35th annual meeting of the Japanese Society for Radiation Oncology (JASTRO), and some results of this study are available in Japanese on the official website of the Japanese Society for Radiation Oncology.
