# Supplementary material for: Multi-institutional prospective observational study of radiotherapy for metastatic bone tumor
Source: J Radiat Res. 2024 Aug 20;65(5):701–11. doi: 10.1093/jrr/rrae060 (PMC11420848; doi:10.1093/jrr/rrae060)
Supplement: supplemental_table_1_rrae060 [file supplemental_table_1_rrae060.docx]

Supplemental Table 1　Irradiated dose and PS

|  |  | Radiation method and BED | | |  |  |  |
| --- | --- | --- | --- | --- | --- | --- | --- |
| PS | N (%) | conventional, < 20 Gy | conventional, 20 Gy <=,  < 30 Gy | conventional, 30 Gy<=, <40 Gy | conventional, <= 40 Gy | SBRT/IMRT, < 50 Gy | SBRT/IMRT, >= 50 Gy |
| 0 | 52 | 8 (15) | 14 (27) | 14 (27) | 5 (10) | 5 (10) | 6 (12) |
| 1 | 86 | 17 (20) | 17 (20) | 38 (44) | 7 (8) | 0 (0) | 7 (8) |
| 2 | 50 | 17 (34) | 10 (20) | 19 (38) | 2 (4) | 0 (0) | 2 (4) |
| 3 | 28 | 9 (32) | 7 (25) | 10 (36) | 2 (7) | 0 (0) | 0 (0) |
| 4 | 8 | 4 (50) | 2 (25) | 2 (25) | 0 (0) | 0 (0) | 0 (0) |

PS: performance status

SBRT: stereotactic body radiotherapy

IMRT: intensity-modulated radiation therapy

BED: biologically effective dose
