# Supplementary material for: Multi-institutional prospective observational study of radiotherapy for metastatic bone tumor
Source: J Radiat Res. 2024 Aug 20;65(5):701–11. doi: 10.1093/jrr/rrae060 (PMC11420848; doi:10.1093/jrr/rrae060)
Supplement: Supplemental_Table2_rrae060 [file supplemental_table2_rrae060.docx]

Supplemental Table 2. Pain relief and opioid dose reduction in patients using opioids at baseline

| pain relief, N (%) | 2-month (N=68) | | 6-month (N=41) | |
| --- | --- | --- | --- | --- |
| Complete Pain Resolution | 13 | 19.1 | 8 | 19.5 |
| opioid dose reduction >= 25% | 11 | 16.2 | 8 | 19.5 |
| opioid dose reduction < 25% | 2 | 2.9 | 0 | 0 |
| Partial Pain Relief | 19 | 27.9 | 12 | 29.3 |
| opioid dose reduction >= 25% | 14 | 20.6 | 11 | 26.8 |
| opioid dose reduction < 25% | 5 | 7.4 | 1 | 2.4 |
| Unchanged | 30 | 44.1 | 17 | 41.5 |
| opioid dose reduction >= 25% | 1 | 1.5 | 0 | 0 |
| opioid dose reduction < 25% | 29 | 42.6 | 17 | 41.5 |
| Worsening | 6 | 8.8 | 4 | 9.8 |
| opioid dose reduction >= 25% | 0 | 0 | 0 | 0 |
| opioid dose reduction < 25% | 6 | 8.8 | 4 | 9.8 |
