# Supplementary material for: Multi-institutional prospective observational study of radiotherapy for metastatic bone tumor
Source: J Radiat Res. 2024 Aug 20;65(5):701–11. doi: 10.1093/jrr/rrae060 (PMC11420848; doi:10.1093/jrr/rrae060)
Supplement: Supplemental_Table_3_rrae060 [file supplemental_table_3_rrae060.docx]

Supplemental Table 3　Details of patients with SRE

| Age | Primary site | sex | PS | Ambulatory status | RT method | RT lesion 1 | BED (Gy) | RT lesion 2 | BED (Gy) | RT lesion 3 | BED (Gy) | SINS | Mirels score | survival  (day) | status | SRE after RT | | | |
| --- | --- | --- | --- | --- | --- | --- | --- | --- | --- | --- | --- | --- | --- | --- | --- | --- | --- | --- | --- |
|  |  |  |  |  |  |  |  |  |  |  |  |  |  |  |  | pathological  fracture | paralysis | surgery | re-irradiation |
| 70 | Renal/ureteral | Female | 1 | Ambulatory | 3DCRT | thoracic spine | 46.9 | femoral bone | 50.7 | pelvic bone | 50.7 | 6 | 9 | 180 | censored | + | - | - | - |
| 81 | Renal/ureteral | Male | 1 | Ambulatory | 3DCRT | femoral bone | 50.7 |  | NA |  | NA | NA | 8 | 213 | censored | + | - | + | - |
| 73 | Lung | Female | 4 | Not Ambulatory | 3DCRT | lumbar spine | 14.4 | thoracic spine | 14.4 | pelvic bone | 14.4 | 10 | NA | 183 | censored | - | - | - | + |
| 72 | Lung | Male | 1 | Ambulatory | 3DCRT | skull bone | 14.4 |  | NA |  | NA | NA | NA | 183 | censored | - | - | - | + |
| 51 | Lung | Male | 0 | Ambulatory | 3DCRT | humerus | 28.0 |  | NA |  | NA | NA | NA | 78 | censored | - | - | + | - |
| 75 | Breast | Female | 2 | In room | 3DCRT | pelvic bone | 14.4 |  | NA |  | NA | NA | NA | 156 | censored | - | - | - | + |
| 67 | Lung | Male | 1 | Ambulatory | 3DCRT | humerus | 33.6 |  | NA |  | NA | NA | NA | 189 | censored | - | - | - | + |
| 59 | Hepatobiliary/pancreatic | Male | 1 | In room | 3DCRT | rib | 14.4 |  | NA |  | NA | NA | NA | 87 | death | + | - | - | - |
| 65 | Lung | Male | 1 | Ambulatory | 3DCRT | thoracic spine | 39.0 |  | NA |  | NA | 7 | NA | 177 | censored | - | - | - | + |
| 82 | Lung | Male | 2 | Not Ambulatory | 3DCRT | cervical spine | 28.0 |  | NA |  | NA | 16 | NA | 204 | censored | - | - | - | + |
| 53 | Uterus | Female | 2 | In room | 3DCRT | pelvic bone | 28.0 |  | NA |  | NA | NA | NA | 189 | censored | + | - | + | - |
| 64 | Lung | Male | 1 | Ambulatory | 3DCRT | rib | 39.0 |  | NA |  | NA | NA | NA | 192 | censored | - | - | - | + |
| 64 | Colon | Male | 2 | In room | 3DCRT | thoracic spine | 14.4 |  | NA |  | NA | 13 | NA | 59 | death | - | + | + | - |
| 52 | Lung | Female | 2 | In room | 3DCRT | femoral bone | 14.4 |  | NA |  | NA | NA | 8 | 98 | death | - | - | - | + |

PS: performance status

RT: radiotherapy

SINS: surgical instability neoplastic score

SRE: skeletal-related event

NA: not applicable

* Data have not been collected on which site SREs occurred when they occurred in patients who were irradiated in multiple sites.
